# Design and Compressive Characterization of Developed Lattice Structures for Lightweight Applications: A Preliminary Study

**DOI:** 10.3390/ma19194067

**Published:** 2026-09-23

**Authors:** Sungmin Kim, Kangbin Cho, Thi Huyeon Vu, Seungyun Lee, Seong Je Park

**Affiliations:** 1 School of Mechanical Engineering, Gyeongsang National University, 501 Jinju-daero, Jinju-si 52828, Gyeongsangnam-do, Republic of Korea; 2 Graduate School of Aerospace and Defense Convergence, Gyeongsang National University, 501 Jinju-daero, Jinju-si 52828, Gyeongsangnam-do, Republic of Korea

**Keywords:** additive manufacturing, material extrusion, lattice structure, lightweight, compressive behavior

## Abstract

Lattice structures offer high lightweight efficiency and tunable mechanical performance, making them promising for applications requiring effective load bearing and controlled deformation. In this study, two new lattice architectures, namely the Hexagonal hub lattice structure (HH) and P-surface-based circular junction lattice structure (PCJ), were proposed, and their compressive behaviors were experimentally investigated. A conventional octet-truss lattice structure (OT) and body-centered cubic lattice structure (BCC) were employed as representative stretch- and bending-dominated references, respectively. The hourglass re-entrant lattice structure (HR) was additionally included for comparison because of its distinctive compressive deformation characteristics. To ensure a consistent comparison, all architectures were designed with an identical unit-cell size and approximately equal material volume and fabricated from polylactic acid using material extrusion. Compression tests revealed distinct load-bearing and deformation characteristics depending on the lattice geometry. HH and PCJ exhibited stretch-dominated-like characteristics comparable to OT and showed relatively high compressive strengths. Among the investigated architectures, HH exhibited the highest compressive strength while maintaining a relatively stable post-peak response. Deformation observations further revealed diagonal shear-band-like deformation in HH, PCJ, and OT, whereas HR exhibited inward lateral contraction associated with its re-entrant geometry and relatively early densification. BCC underwent progressive layer-by-layer collapse without pronounced diagonal localization. These results demonstrate that tailoring lattice geometry can effectively control load-transfer and collapse mechanisms, providing a design strategy for lightweight structures requiring high compressive load-bearing capability and controlled deformation behavior.

## 1. Introduction

Additive manufacturing (AM) has emerged as an effective manufacturing technology for producing complex three-dimensional structures that are difficult to fabricate using conventional manufacturing processes [1,2,3]. In particular, the layer-by-layer nature of AM enables a high degree of geometric freedom, allowing for the fabrication of lightweight structures with complex internal architectures [4,5,6]. Among various AM technologies, material extrusion (MEX) has been widely adopted because of its relatively low equipment cost, simple operation, and compatibility with a broad range of thermoplastic materials [7,8,9]. These advantages make MEX particularly suitable for the rapid fabrication and experimental evaluation of geometrically complex structures [10,11].

Lattice structures have attracted considerable attention as one of the representative applications of the geometric freedom provided by AM [12,13,14,15]. They consist of periodically arranged unit cells and can provide high specific stiffness and strength while maintaining low structural weight [16,17]. In addition, their mechanical response can be tailored by modifying geometric parameters such as unit-cell topology, member orientation, connectivity, and relative density [18,19,20]. Owing to these characteristics, lattice structures have been investigated for lightweight structural components, impact protection, energy absorption, and aerospace applications, including landing systems in which structures are required to withstand compressive and impact loads while minimizing overall weight [21,22].

The compressive behavior of lattice structures is strongly governed by their topology and the resulting load-transfer mechanism. In general, lattice architectures can exhibit stretch-dominated or bending-dominated deformation depending on the connectivity and orientation of their constituent members [23,24,25]. Stretch-dominated architectures primarily transfer external loads through axial deformation of their members and therefore generally exhibit relatively high stiffness and load-bearing capacity [26,27,28]. In contrast, bending-dominated architectures accommodate deformation primarily through member bending and rotation, generally resulting in lower stiffness and strength but more progressive deformation and a relatively stable plateau region [29,30,31]. These differences also affect the subsequent collapse behavior, including localized deformation, sequential collapse, and densification. Therefore, controlling the dominant deformation mechanism through geometric design is essential for tailoring the compressive performance of lattice structures.

Various lattice architectures have been developed to achieve specific mechanical characteristics. Among them, the octet-truss (OT) is widely recognized as a representative stretch-dominated architecture, in which external loads are primarily supported by axial tensile and compressive stresses in the constituent struts, resulting in relatively high stiffness and strength [32,33]. In contrast, the body-centered cubic (BCC) architecture typically exhibits bending-dominated behavior, providing progressive deformation under compression [34,35]. In addition, triply periodic minimal surface (TPMS)-inspired geometries have been explored to introduce smooth geometric transitions and continuous load-transfer paths [36,37]. Recent studies on metallic lattice structures have further demonstrated the significant influence of lattice geometry on compressive performance and deformation behavior. In AlSi10Mg lattices, strut-based and TPMS-based architectures exhibited substantially different mechanical properties and energy absorption characteristics depending on the unit-cell topology, with distinct stretch- and bending-dominated deformation behaviors [38]. Similarly, powder bed fusion-fabricated Ti-6Al-4V lattice structures developed through topology optimization showed that geometric configuration and material distribution strongly affected compressive strength and stress distribution [39]. Comparative investigations of Inconel 625 and Ti-6Al-4V strut lattices also demonstrated that unit-cell topology strongly influences compressive performance across different metallic materials [40]. Furthermore, a recent investigation of AA6082 TPMS cellular structures showed that relative density significantly affected collapse behavior. The lower-density structure exhibited progressive and distributed collapse with an extended plateau region, whereas the higher-density structure showed increased load-bearing capacity but more localized collapse and earlier densification [41]. Collectively, these studies indicate that geometric configuration plays a critical role not only in strength and stiffness but also in deformation and collapse mechanisms of metallic lattice structures.

Despite extensive research on conventional lattice architectures, the development of new lattice geometries remains important for achieving combinations of high load-bearing capacity and controlled deformation behavior. Conventional stretch-dominated architectures can provide high compressive strength but may undergo pronounced stress drops associated with localized or sequential collapse after reaching the initial peak load. Conversely, bending-dominated architectures generally provide more progressive deformation but at the expense of reduced load-bearing capacity. Therefore, designing lattice architectures capable of combining efficient load transfer with stable post-yield deformation remains an important challenge. Furthermore, comparative evaluation under equivalent material-volume conditions is necessary to distinguish the effects of lattice topology from those arising simply from differences in the amount of constituent material [25,42].

In this study, two lattice architectures, namely the hexagonal hub lattice structure (HH) and P-surface-based circular junction lattice structure (PCJ), were proposed to investigate the influence of geometric design on compressive performance. The novelty of this study lies in the development of these two architectures and their systematic comparison with conventional lattice structures under equivalent material-volume conditions. For comparison, OT, BCC, and hourglass re-entrant lattice structure (HR) were selected as representative conventional architectures exhibiting stretch-dominated, bending-dominated, and distinctive re-entrant deformation characteristics, respectively. To eliminate the influence of material quantity, the material volume of all architectures was maintained at approximately 20,000 mm^3^. The specimens were fabricated using MEX and experimentally evaluated under quasi-static compression. The compressive strength, stress–strain response, and deformation and collapse behavior were comparatively analyzed to clarify the relationship between lattice geometry, dominant load-transfer mechanism, and compressive performance. The findings provide a geometric design basis for developing lightweight lattice structures with tailored load-bearing and deformation characteristics for applications subjected to compressive loading.

## 2. Methodology

### 2.1. Lattice Structure Design

All lattice structures were designed using Autodesk Inventor Professional (Autodesk Inc., San Francisco, CA, USA). To ensure a consistent comparison among the proposed lattice architectures, a unit cell size of 8 mm × 8 mm × 8 mm was adopted for all designs, as shown in Figure 1. To eliminate the influence of relative density, the material volume of each CAD model was maintained at approximately 20,000 mm^3^ by adjusting the beam thickness of each lattice architecture. Previous studies have demonstrated that lattice specimens containing at least four unit cells along each loading direction exhibit size-independent mechanical properties [43]. Therefore, each specimen was constructed by periodically repeating five unit cells along the x-, y-, and z-directions, resulting in an overall specimen dimension of 40 mm × 40 mm × 40 mm. Two lattice architectures were subsequently developed based on different geometric design concepts inspired by traditional Korean cultural motifs and engineering principles. The primary design criteria were geometric symmetry, continuous member connectivity, manufacturability by MEX, and the formation of distinct load-transfer paths under compression. The detailed design rationale for each lattice structure is described in Section 2.1.1 and Section 2.1.2.

#### 2.1.1. Hexagonal Hub Lattice Structure (HH)

The HH was inspired by the geometric configuration of Hyangwonjeong Pavilion in Gyeongbokgung Palace, one of the representative traditional Korean pavilions characterized by its highly symmetric hexagonal floor plan and centralized architectural layout [44]. Rather than directly reproducing the architectural appearance, the essential geometric features, including the central hexagonal region and the surrounding enclosed perimeter, were extracted and reconstructed into a manufacturable lattice architecture suitable for MEX. The proposed design consists of a central hexagonal hub connected to an outer circular-inspired frame through four primary supporting members, resulting in a highly symmetric structural configuration, as shown in Figure 1a.

#### 2.1.2. P-Surface-Based Circular Junction Lattice Structure (PCJ)

The PCJ lattice was inspired by the geometric patterns of traditional Korean wooden lattice-window structures (Munsal), which are characterized by repetitive square-lattice arrangements that provide both structural support and aesthetic harmony in traditional Korean architecture [45]. The proposed lattice consists of a square peripheral frame interconnected through circular junctions, resulting in a symmetric structural configuration with continuous geometric connectivity. From an engineering perspective, the circular junctions were further refined based on the geometric transition concept of the Schwarz P minimal surface [46]. Instead of employing sharp intersections between adjacent members, smoothly curved transition regions were incorporated to establish continuous connections between the surrounding structural members. Such geometric continuity was intended to reduce abrupt changes in structural stiffness and promote a more uniform transfer of compressive loads throughout the lattice. It should be noted that the proposed geometry is not a TPMS lattice but rather a simplified lattice architecture inspired by the geometric characteristics of the Schwarz P minimal surface, as shown in Figure 1b.

#### 2.1.3. Reference Lattice Structures (OT, BCC, and HR)

As shown in Figure 1c–e, to evaluate the mechanical performance of the proposed lattice architectures, three widely used conventional lattice structures, namely the OT, BCC, and HR were selected as reference structures. The OT is a representative stretch-dominated topology, in which the applied load is primarily transferred through axial deformation of the struts, resulting in relatively high stiffness and strength [4]. In contrast, the BCC is generally regarded as a bending-dominated topology, where the inclined struts predominantly deform by bending under compressive loading, leading to progressive deformation and enhanced energy absorption [29]. The HR was additionally included because of its distinctive deformation behavior under compression, characterized by inward lateral contraction associated with its re-entrant geometry [47]. By comparing the proposed HH and PCJ architectures with these conventional reference structures, the influence of the newly developed geometries on compressive behavior could be systematically evaluated.

### 2.2. Fabrication of Lattice Structures via MEX

A commercially available PLA filament (Hanil Industry Co., Ltd., Seoul, Republic of Korea) with a diameter of 1.75 mm was employed as the feedstock material in this study. PLA was selected because of its excellent processability and widespread use in MEX. All specimens were fabricated using a commercially available MEX system (Bambu Lab A1, Bambu Lab, Shenzhen, China). A nozzle with a diameter of 0.4 mm was used, and the nozzle and build plate temperatures were set to 250 °C and room temperature, respectively. The raster angle was set to ±45°, the layer thickness was 0.20 mm, and the nozzle travel speed was 150 mm/s. The infill density was fixed at 100%. All lattice structures were fabricated using identical processing parameters to ensure a fair comparison of their compressive mechanical performance.

### 2.3. Compressive Testing

The compressive properties of the fabricated lattice structures were evaluated using a universal testing machine (AGX-10kNVD, Shimadzu Corp., Kyoto, Japan) equipped with a 10 kN load cell. Compression tests were performed under quasi-static loading at a crosshead speed of 5 mm/min until densification of the lattice structures. The compressive load and displacement were continuously recorded throughout the test, and the engineering stress–strain curves were subsequently calculated. Three specimens were tested for each lattice architecture, and the average values are reported.

## 3. Results

### 3.1. Compressive Properties

Figure 2a shows the compressive stress–strain curves of HH, PCJ, OT, BCC, and HR. Distinct compressive responses were observed depending on the lattice geometry. In particular, HH, PCJ, and OT exhibited characteristics associated with stretch-dominated deformation, whereas BCC and HR showed characteristics more closely associated with bending-dominated deformation.

OT, which is generally regarded as a representative stretch-dominated architecture, exhibited a rapid increase in stress during the initial stage of compression and reached a relatively high initial peak stress. In stretch-dominated deformation, the applied compressive load is primarily transferred through axial deformation of the constituent members, generally resulting in relatively high stiffness and load-bearing capacity. After reaching the initial peak, OT showed a distinct stress drop followed by repeated increases and decreases in stress, resulting in a serrated stress–strain response. This behavior indicates progressive and sequential collapse of the constituent members during compression [48,49,50]. PCJ exhibited a similar response, characterized by a relatively high initial peak stress followed by repeated stress fluctuations. The similarity between PCJ and OT suggests that axial load transfer contributes substantially to the compressive behavior of PCJ, resulting in stretch-dominated-like characteristics [51]. HH also exhibited characteristics associated with stretch-dominated load transfer, as reflected by its relatively high initial load-bearing capacity [52]. However, its post-peak behavior was clearly different from those of OT and PCJ. While OT and PCJ exhibited pronounced stress drops and serrated responses after the initial peak, HH maintained a relatively stable stress level without substantial fluctuations over a wide strain range. The stress subsequently increased as densification progressed. This indicates that HH can maintain the high load-bearing capability associated with stretch-dominated deformation while exhibiting a more stable post-peak response. The highly interconnected and symmetric geometry of HH may contribute to effective load transfer and suppress abrupt sequential collapse during compression [53].

In contrast, BCC and HR exhibited characteristics associated with bending-dominated deformation. BCC, which is generally regarded as a representative bending-dominated architecture, showed a relatively low initial stress followed by a broad plateau region. In bending-dominated deformation, the constituent members primarily accommodate the applied load through bending rather than axial deformation, generally resulting in a lower initial load-bearing capacity and more progressive deformation [19,23]. HR exhibited a similar tendency, with a relatively lower initial load-bearing response and a well-developed plateau region over a wide strain range. This behavior is consistent with the re-entrant geometry of HR, in which the inclined members promote bending and rotation during compression. Therefore, the stress–strain responses suggest that HH and PCJ exhibit predominantly stretch-dominated-like characteristics comparable to OT, whereas BCC and HR exhibit greater contributions from bending-dominated deformation.

These differences in compressive behavior are further reflected in the compressive strength results shown in Figure 2b. HH, PCJ, and OT, which exhibited stretch-dominated-like characteristics, showed relatively high compressive strengths, whereas BCC and HR, which exhibited greater contributions from bending-dominated deformation, showed comparatively lower compressive strengths. Among the five architectures, HH exhibited the highest compressive strength while also maintaining a relatively stable post-peak response. These results demonstrate that the dominant deformation mechanism governed by lattice structure geometry plays an important role in determining both the compressive strength and deformation characteristics.

For broader comparison, conventional two-dimensional cellular structures, such as hexagonal, triangular, circular, and square honeycomb-like architectures, generally exhibit strongly direction-dependent compressive behavior [54]. Under out-of-plane compression, their cell walls primarily undergo axial compression and buckling, resulting in relatively high load-bearing capability, whereas in-plane compression is predominantly governed by cell-wall bending and sequential collapse, leading to lower stiffness and strength [55]. In contrast, the three-dimensional lattice architectures investigated in the present study provide spatially interconnected load-transfer paths, resulting in architecture-dependent responses ranging from stretch-dominated-like behavior in HH, PCJ, and OT to bending-dominated deformation in BCC and re-entrant deformation in HR. However, because the constituent materials, relative densities, geometric dimensions, and fabrication conditions differ among the reported studies, a direct quantitative comparison of compressive strength is not appropriate. Nevertheless, this comparison demonstrates the distinct load-transfer and deformation characteristics achievable through three-dimensional lattice architectures compared with conventional two-dimensional cellular configurations.

### 3.2. Compressive Deformation Behavior

Figure 3 shows the deformation behavior of HH, PCJ, OT, BCC, and HR during compression. The images on the left, middle, and right show the specimens before compression, during compression prior to yielding, and after reaching the yield stress, respectively. The intermediate deformation states were included to more clearly illustrate how each lattice structure deformed and collapsed during compression. Comparison of the deformation states revealed distinct collapse modes depending on the geometric configuration of each architecture.

HH, PCJ, and OT, which exhibited stretch-dominated-like characteristics in Figure 2a, showed localized deformation along a diagonal direction during compression. As indicated by the dashed lines in Figure 3a–c, deformation was concentrated within an inclined band oriented approximately 45° to the loading direction. This diagonal shear-band-like deformation indicates that, after the initial load-bearing stage dominated by axial load transfer, structural instability became localized along a preferential diagonal path [29,56,57,58]. The formation and propagation of the localized collapse band resulted in progressive loss of load-bearing members, consistent with the post-peak behavior observed in the stress–strain curves. In particular, the pronounced stress fluctuations observed for PCJ and OT can be associated with successive local collapse events as the deformation band propagated through the architectures. HH also developed a diagonal deformation band; however, its stress–strain response remained comparatively stable after the initial peak, suggesting that load redistribution during localized deformation occurred more continuously than in PCJ and OT.

BCC exhibited another distinct collapse mechanism, as shown in Figure 3d. Unlike the diagonal localization observed for HH, PCJ, and OT, BCC underwent progressive compression without the formation of a pronounced shear band. Instead, the unit-cell layers collapsed sequentially along the loading direction, resulting in a relatively uniform layer-by-layer deformation mode. Similar progressive collapse without pronounced shear-band formation has also been reported for BCC under compression [59,60]. This progressive collapse is consistent with the bending-dominated characteristics of BCC and explains its broad plateau region and relatively low stress level in Figure 2a. As compression continued, the internal voids were gradually closed, and contact between the collapsed members increased, eventually leading to densification and the corresponding rapid increase in stress.

HR exhibited a markedly different deformation mode, as shown in Figure 3e. Rather than developing a distinct diagonal collapse band, HR underwent inward lateral contraction during axial compression. HR is a conventional re-entrant architecture in which rotation and bending of the inclined members can induce inward lateral contraction during axial compression [61,62]. This inward contraction reduces the internal void space and promotes earlier contact between adjacent members. Consequently, densification occurred at a relatively small strain, consistent with the earlier rapid increase in stress observed for HR in Figure 2a.

Overall, the deformation observations in Figure 3 support the mechanical responses identified from the stress–strain curves. HH, PCJ, and OT exhibited stretch-dominated-like load-bearing characteristics followed by localized diagonal collapse, whereas BCC exhibited progressive layer-by-layer collapse characteristic of bending-dominated deformation. HR exhibited inward lateral contraction associated with its re-entrant geometry and relatively early densification. These results demonstrate that lattice geometry governs not only the initial load-bearing capability but also the localization, progression, and densification mechanisms during compression.

## 4. Discussion

The present results demonstrate that lattice geometry can be used to tailor compressive behavior even when the material volume and overall specimen dimensions are maintained at approximately identical levels. From a structural design perspective, compressive strength and deformation behavior should be considered together when selecting a lattice architecture. A geometry providing high load-bearing capacity does not necessarily exhibit stable deformation after the initial peak, whereas a geometry showing progressive deformation may provide a lower maximum compressive strength. Therefore, lattice topology should be selected according to the mechanical requirements of the intended application rather than solely on the basis of compressive strength. The proposed HH and PCJ architectures provide different geometric approaches for controlling these mechanical characteristics. In particular, the behavior of HH suggests that its highly interconnected and symmetric configuration can provide high load-bearing capability together with relatively stable post-peak deformation. PCJ also provides relatively high load-bearing capability, although its post-peak response indicates more pronounced localized collapse. These behaviors can be interpreted in relation to the conventional reference structures, with OT providing a representative stretch-dominated response and BCC exhibiting progressive bending-dominated deformation. In comparison, the conventional re-entrant HR architecture exhibits progressive deformation through member bending and inward lateral contraction. These differences demonstrate that modification of member connectivity and geometric configuration can provide different balances between load-bearing capability and deformation stability, providing a basis for further development of lightweight lattice structures. Nevertheless, the present study should be regarded as a preliminary comparative investigation under controlled experimental conditions. Only a single material volume and unit-cell size were considered, and all specimens were fabricated from PLA using fixed MEX processing parameters. In addition, the mechanical evaluation was limited to quasi-static compression in a single loading direction. Further investigation of relative density, geometric scale, constituent materials, and different loading conditions, together with numerical analysis and systematic parametric studies, will therefore be necessary to determine the generality of the observed behaviors and to establish design guidelines for practical applications.

## 5. Conclusions

In this study, two lattice architectures, HH and PCJ, were proposed and fabricated using MEX, and their compressive properties and deformation behaviors were comparatively evaluated against those of three conventional reference structures, OT, BCC, and HR. To ensure a consistent comparison, all architectures were designed with approximately identical material volumes and tested under the same compression conditions. The main conclusions are summarized as follows.

(1) HH and PCJ exhibited stretch-dominated-like characteristics comparable to OT, characterized by relatively high initial load-bearing capacities and compressive strengths. In contrast, BCC exhibited representative bending-dominated characteristics, including a relatively low initial load-bearing capacity and a broad plateau region, while HR showed a greater contribution from bending-dominated deformation.
(2) Among the five architectures, HH exhibited the highest compressive strength while maintaining a relatively stable post-peak response. Unlike PCJ and OT, which showed pronounced stress fluctuations after the initial peak, HH maintained its load-bearing capability without substantial fluctuations, demonstrating a favorable combination of high compressive strength and stable deformation.
(3) Distinct collapse mechanisms were observed depending on the lattice geometry. HH, PCJ, and OT developed localized diagonal shear-band-like deformation at approximately 45° to the loading direction. BCC underwent progressive layer-by-layer collapse without pronounced diagonal localization, whereas HR exhibited inward lateral contraction associated with its re-entrant geometry, which promoted earlier contact between adjacent members and relatively early densification.

Overall, the results demonstrate that lattice geometry plays a critical role in governing the load-transfer mechanism, compressive strength, post-peak response, and collapse behavior. By introducing HH and PCJ and systematically comparing them with conventional reference structures under equivalent material-volume conditions, this study demonstrates how geometric design can be used to tailor the balance between load-bearing capability and deformation behavior. In particular, HH provided high load-bearing capability together with stable post-peak deformation, highlighting its potential as a lightweight lattice architecture for applications requiring compressive load resistance. These findings provide a basis for further optimization and evaluation of the proposed HH and PCJ architectures under application-specific loading conditions.

## Figures and Tables

**Figure 1:**
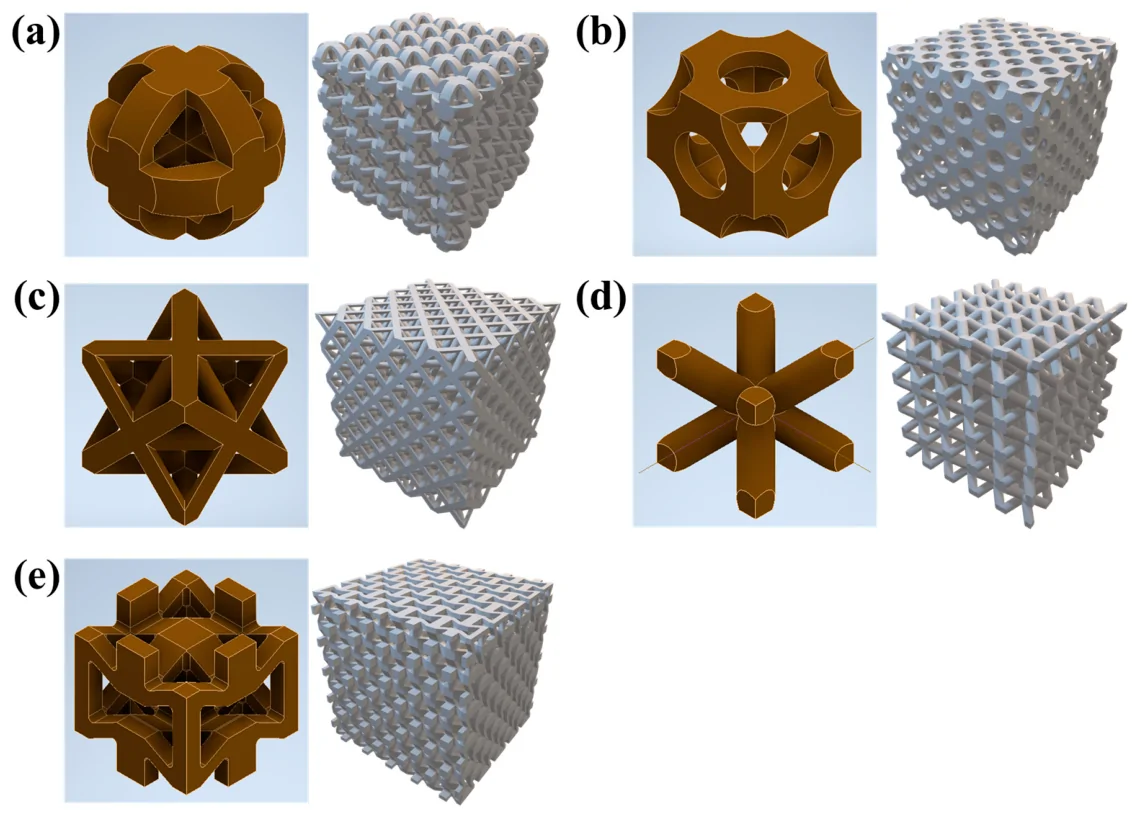
Schematic illustration and CAD models of the lattice architectures: (**a**) HH, (**b**) PCJ, (**c**) OT, (**d**) BCC, and (**e**) HR.

**Figure 2:**
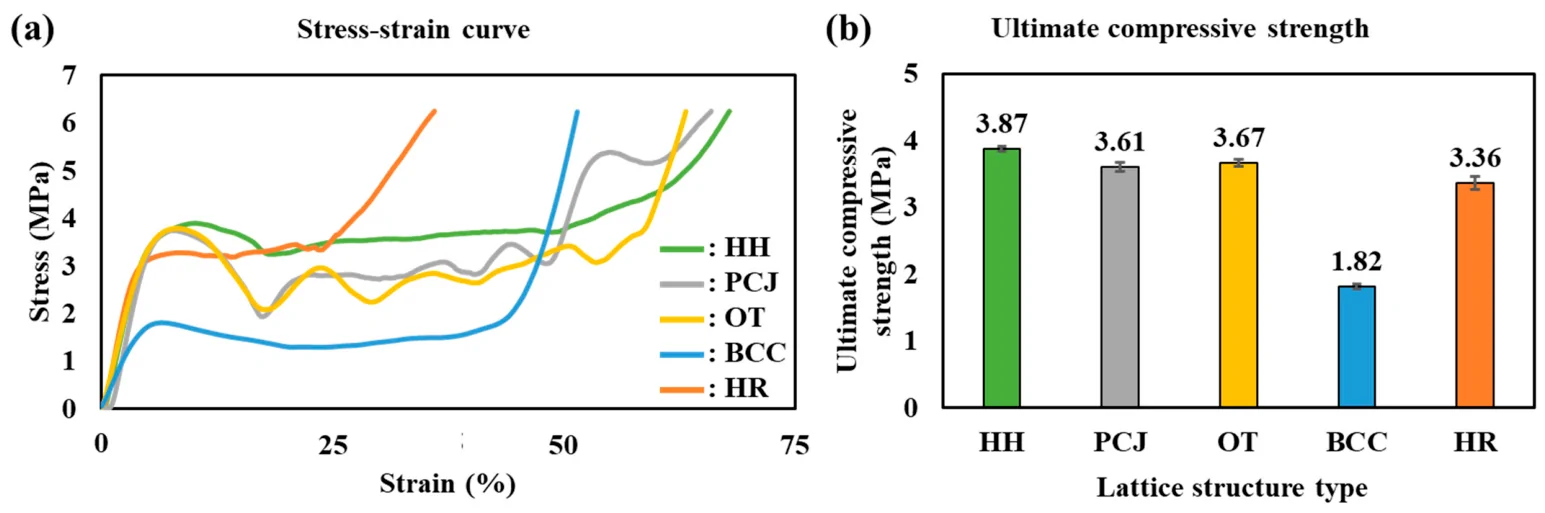
Compressive properties of HH, PCJ, OT, BCC, and HR: (**a**) compressive stress–strain curves and (**b**) ultimate compressive strength (n = 3).

**Figure 3:**
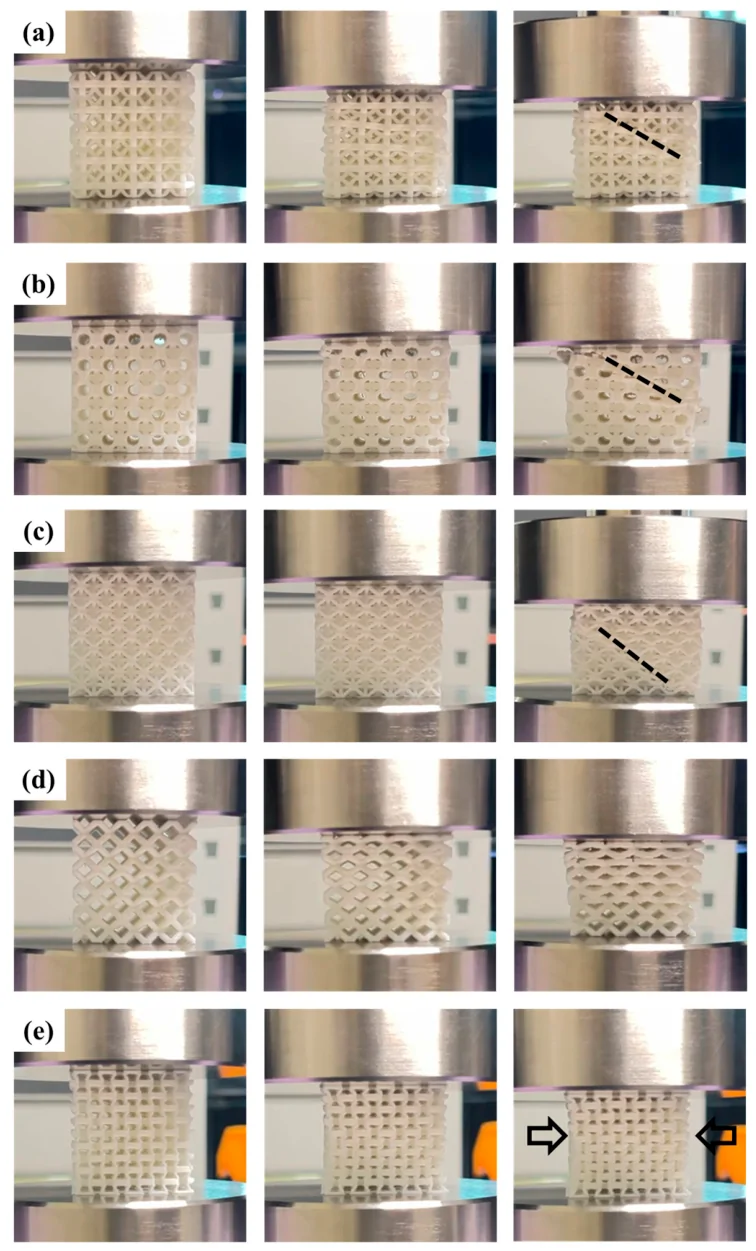
Compressive deformation behavior of (**a**) HH, (**b**) PCJ, (**c**) OT, (**d**) BCC, and (**e**) HR. The left, middle, and right images show the specimens before compression, during compression, and after reaching the yield stress, respectively. Dashed lines indicate localized diagonal deformation bands, and arrows indicate the inward lateral contraction of HR.

## Data Availability

The original contributions presented in this study are included in the article. Further inquiries can be directed to the corresponding author.

## Abbreviations

AM	Additive manufacturing
BCC	Body-centered cubic lattice structure
CAD	Computer-aided design
HH	Hexagonal hub lattice structure
HR	Hourglass re-entrant lattice structure
MEX	Material extrusion
OT	Octet-truss lattice structure
PCJ	P-surface-based circular junction lattice structure
PLA	Polylactic acid
TPMS	Triply periodic minimal surface
